# The comparison between intratympanic gentamicin prehabilitation and postoperative virtual reality exposure to standard vestibular training in patients with vestibular schwannoma

**DOI:** 10.1007/s00405-024-08891-8

**Published:** 2024-08-10

**Authors:** Markéta Bonaventurová, Zuzana Balatková, Květoslav Červený, Rudolf Černý, Veronika Bandúrová, Vladimír Koucký, Lenka Peterková, Zdeněk Fík, Martin Komarc, Eva Mrázková, Jan Plzák, Zdeněk Čada

**Affiliations:** 1 Department of Otorhinolaryngology and Head and Neck Surgery, 1st Faculty of Medicine, Charles University in Prague and Motol University Hospital, V Úvalu 84, Prague, 150 06 Czech Republic; 2 Department of Neurology, 2nd Faculty of Medicine, Charles University, Motol University Hospital, Prague, Czech Republic; 3 Department of Anthropomotorics and Methodology, Faculty of Physical Education and Sport, Charles University, Prague, Czech Republic; 4 Department of Otorhinolaryngology, The Second Faculty of Medicine, Charles University, University Hospital Motol, Prague, Czech Republic; 5 Department of Otorhinolaryngology and Head and Neck Surgery, Havířov Hospital, Havířov, Czech Republic

**Keywords:** Vestibular schwannoma, Virtual reality, Prehabilitation, Intratympanic gentamicin, Optokinetic tests, Video head impulse test

## Abstract

**Objective:**

Resection of the vestibular schwannoma causes acute peripheral vestibular loss. The process of central compensation starts immediately afterward. The rehabilitation goal is to support this process and restore the quality of life.

**Materials and methods:**

In this prospective single-center study, 67 consecutive patients underwent vestibular schwannoma resection (40 females, mean age 52 ± 12 years). The patients were divided into three groups: the prehabilitation with intratympanic gentamicin group, the virtual reality group (optokinetic stimulation via virtual reality goggles in the first ten days after the surgery), and the control group. All patients were examined with objective methods and completed questionnaires before the prehabilitation, before the surgery, at the hospital discharge, and after three months.

**Results:**

Intratympanic gentamicin prehabilitation leads ipsilaterally to a significant aVOR reduction in all semicircular canals (*p* < 0.050), the increase of the unilateral weakness in air calorics (*p* = 0.026), and loss of cVEMPs responses (*p* = 0.017). Prehabilitation and postoperative exposure to virtual reality scenes improved the patient’s perception of vertigo problems according to Dizziness Handicap Inventory (*p* = 0.039 and *p* = 0.076, respectively). These findings conform with the optokinetic testing results, which showed higher slow phase velocities at higher speeds (40 deg/s) in both targeted groups compared to the control group.

**Conclusion:**

Preoperative intratympanic gentamicin positively affects peripheral vestibular function, influencing balance perception after VS resection. In long-term follow-up, prehabilitation and postoperative exposure to virtual reality improve patients’ quality of life in the field of vertigo problems.

**Supplementary Information:**

The online version contains supplementary material available at 10.1007/s00405-024-08891-8.

## Introduction

Vestibular schwannoma (VS) is a benign, slow-growing intracranial tumor arising from Schwann cells, which myelinate the vestibular portion of the eighth cranial nerve. It originates in the internal auditory canal and can extend into the cerebellopontine angle (CPA). The incidence of these tumors is 1 per 100.000 persons/year, and the rate has increased over time [1]. The most typical clinical presentations associated with VS are hearing loss, tinnitus, vertigo, dizziness, and postural instability. Diagnosis typically occurs in the fifth or sixth decade of life [1]. Current management options include (i) wait and scan by MRI, (ii) stereotactic radiotherapy with the Leksell gamma knife or CyberKnife, and (iii) surgical resection [2].

As far as the surgical approach is considered, the vestibular branch of the eighth cranial nerve is almost always dissected. Thus, the patient experiences partial or complete unilateral loss of vestibular function. The peripheral vestibular system comprises the saccule, utricle, and semicircular canals. The neuroepithelial hair cells within the peripheral vestibular apparatus send projections to the vestibular nuclei in the caudal pons and rostral dorsolateral medulla by the vestibular division of the eighth cranial nerve. Some nuclei receive only primary vestibular afferents, but most receive afferents from the cerebellum, reticular formation, spinal cord, and contralateral vestibular nuclei. The projections from the vestibular nuclei extend to the cerebellum, extraocular nuclei, and spinal cord [3]. Any perturbation induces static as well as dynamic vestibular imbalance, therefore causing postural (inclination of the head and body toward the affected side, incapacity to stabilize the posture while moving), perceptual (vertigo sensation with the deviation of subjective visual vertical, spatial disorientation) and oculomotor syndrome (spontaneous nystagmus, vertical strabismus, ocular tilt reaction) [4].

The neurologic phenomenon of vestibular compensation is based on central nervous reorganization, leading to functional rehabilitation and recovery. The whole process of central compensation is specific to each patient. Unalterable factors can affect the outcome, such as patient status (age, memory, general physical health, cognitive abilities, vision, neurologic or internal comorbidities, presence of psychological and anxiety disorders) and tumor specification (size and propagation). On the other hand, some factors can be the target of our intervention (vestibular rehabilitation, virtual reality, prehabilitation) [4]. The level of the compensation could be evaluated subjectively with questionnaires (e.g., DHI – dizziness handicap inventory, PANQOL – The Penn acoustic neuroma quality of life scale) or objectively with physical examination (presence of the spontaneous nystagmus, subjective visual vertical) and electrophysiological investigation [5, 6].

Nowadays, we have a battery of tests that can examine each part of the vestibular system separately. For example, videonystagmography (VNG) tests the function of the cerebellum, brain stem, and central nervous system; air calorics tests the lateral semicircular canal and superior vestibular nerve; video head-impulse test (vHIT) evaluates the anterior, lateral, and posterior semicircular canal; cervical vestibular-evoked myogenic potentials (cVEMP) examines predominantly saccule and inferior vestibular nerve; ocular vestibular-evoked myogenic potentials (oVEMP) is predominantly the test for utricular function [7, 8].

The present study compared the efficacy of several types of treatment in patients after vestibular schwannoma surgery.

The primary aim is to compare a traditional patient-customized vestibular rehabilitation program after the surgery to (i) prehabilitation with intratympanic gentamicin before surgery with home-based vestibular training and (ii) a postoperative program based on three-dimensional optokinetic stimulation via virtual reality goggles. We assume both prehabilitation (and preoperative achievement of central compensation) and exposure to visual motion and visuo-vestibular conflict situations in the early state of unilateral peripheral loss after vestibular schwannoma resection can significantly improve postoperative recovery.

## Materials and methods

### Patients

We enrolled 67 consecutive patients from the Department of Otorhinolaryngology and Head and Neck Surgery, 1st Faculty of Medicine Charles University in Prague and Motol University Hospital, who underwent vestibular schwannoma resection between January 2020 and June 2023. The study was approved by the Ethics Committee on Research Projects (EK – 297/20). Due to ethical concerns, the selection of the three groups was not strictly random. Patients with serviceable hearing were randomly divided into a virtual reality group (VRG) or a control group (CG). Patients with non-serviceable hearing and those with larger tumors with brainstem and cranial nerve displacement had the choice to willingly undergo gentamicin treatment (ITGG) or not because the probability of hearing preservation for them was low.

**Fig. 1:**
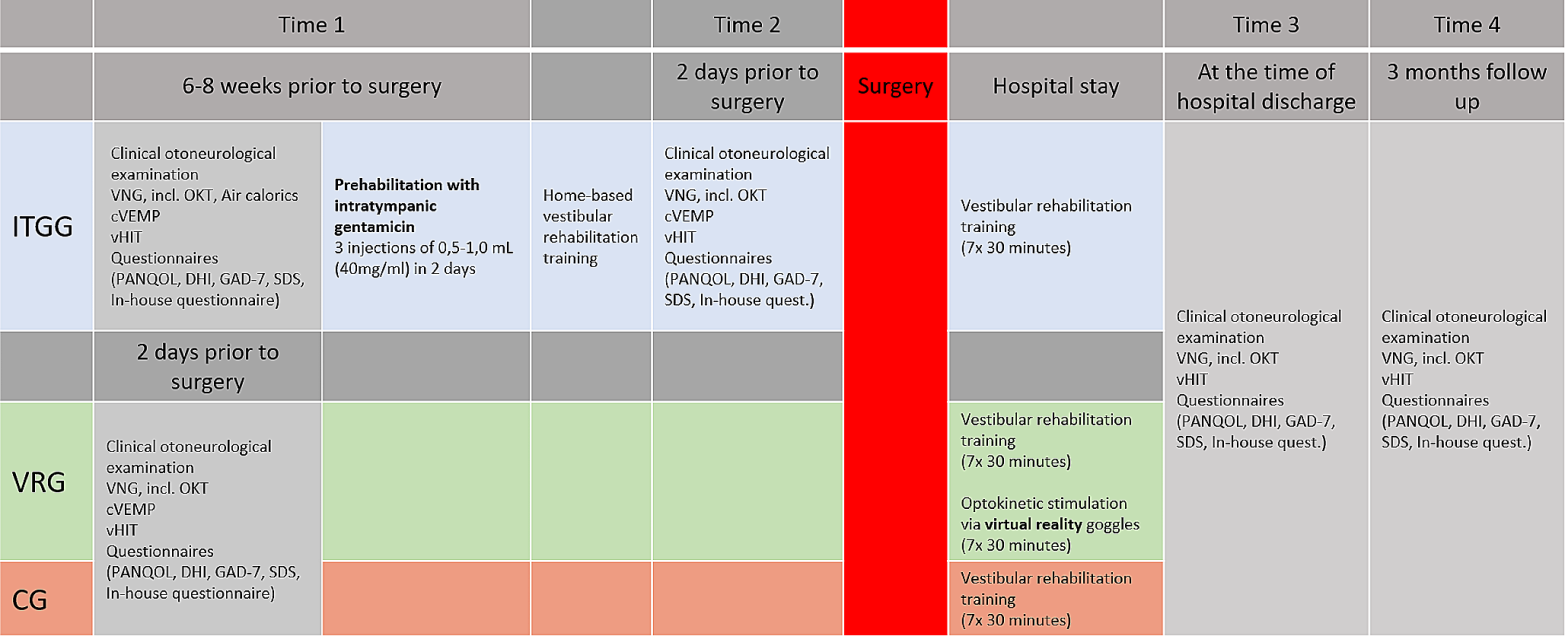
Study flowchart ITGG – Intratympanic Gentamicin Group, VRG – Virtual Reality Group, CG – Control Group, VNG – Videonystagmography, OKT – Optokinetic testing, cVEMP – cervical Vestibular Evoked Myogenic Potentials, vHIT – video Head Impulse Test, PANQOL - Penn acoustic neuroma quality-of-life scale, DHI - Dizziness Handicap Inventory, GAD – 7 - Generalized Anxiety Disorder 7–item scale, SDS - Self-rating Depression Scale

VRG included 26 patients (16 females, mean age 51 ± 12 years), ITGG included 15 patients (11 females, mean age 51 ± 16 years), and CG included 26 patients (14 females, mean age 52 ± 10 years). All surgeries were performed by a multidisciplinary team of neurosurgeons and ear surgeons using mostly lateral suboccipital retrosigmoid approach (*n* = 63,94%) and occasionally translabyrinthine approach (*n* = 4,6%). The tumor size was classified according to the Koos classification [9]. A day before any intervention (time 1), all the patients underwent thorough clinical otoneurological examination and examination by objective methods - VNG including air calorics (AC), vHIT, and cVEMP. They were also required to fill out a set of questionnaires. It included the Penn Acoustic Neuroma Quality-Of-Life scale (PANQOL), Dizziness Handicap Inventory (DHI), Generalized Anxiety Disorder 7–item scale (GAD-7), and Self-rating Depression Scale (SDS). In addition, we used an in–house questionnaire (Table 1) designed by our otoneurologic department based on the most frequent complaints in our clinical practice. The ITGG underwent all these assessments two days before surgery again (time 2). Before the discharge from the hospital (time 3) and in the three-month follow-up (time 4), all patients filled out the same set of questionnaires and underwent, apart from the clinical otoneurologic examination, vHIT and optokinetic testing. The scheme of our study is shown in Fig. 1.

**Table 1:** In–house questionnaire

No.	Question	never	sometimes	very often	always
1	Do you have instability with/or does faster rotational motion bother you (e.g., rotating head from side to side when crossing the road)?				
2	Do you have instability with/or does walking on uneven surfaces bother you (e.g., walking up the stairs/walk in the snow)?				
3	Do you have instability with/or does quickly changing position bother you (e.g., lying on a bed/getting up/recumbent)?				
4	Do you have instability with/or does walking in darkness/twilight bother you?				
5	Do you have instability with/or does reading while driving bother you (the ability to keep eyes fixed when walking)?				
6	Do you have instability with/or does shopping in a supermarket bother you (rapid changes in products on the shelves)?				
7	Do you have instability with/or does a greater amount of auditory and visual sensations bother you (e.g., shopping malls)?				
8	Do you have instability with/or does a longer reading time bother you?				
9	Do you have instability with/or does watching TV bother you?				

### Prehabilitation

All patients in ITGG received treatment with intratympanic gentamicin ipsilaterally to the VS applied by an otolaryngologist 6 to 8 weeks before surgery. They obtained three injections of gentamicin (40 mg/mL) in two days. The dosage ranged between 0.5-1mL depending on the volume of the tympanic cavity. Afterward, the patients were instructed on how to perform home-based vestibular training. Pure tone audiometry (PTA) was obtained in all patients at the baseline and after the surgery. We obtained the PTA six to eight weeks after gentamicin application in only 7 out of 15 patients because it was not the primary aim of our study.

### Virtual reality

In the first ten days after the surgery, VRG obtained seven sessions of 30-minute-long optokinetic stimulation (velocity 20 deg/s-40 deg/s) via Windows Mixed Reality goggles by Acer Inc., Taipei, Taiwan, using software developed by Pro-Zeta a.s., Prague, Czech Republic, and tailored to our needs.

### Vestibular training program

On the second postoperative day, all three groups carried out the patient-customized vestibular training program adopted for patients with acute vestibular loss under the supervision of a physical therapist. The program included gaze stability exercises, smooth pursuit, saccadic eye movements, and postural exercises to improve balance control and gait stability.

Each session lasted 30 min and happened daily for the whole hospitalization, thus a minimum of seven days. The mean length of hospital stay after the surgery was 11 ± 4 days.

### Objective methods

*Videonystagmography* is a complex diagnostic system that can test oculomotor function (saccade, tracking, and optokinetic test), gaze stabilization (gaze/spontaneous nystagmus), and calorization [10]. For our study, we used the ICS Chartr 200 VNG/ENG by Otometrics, Natus, Taastrup, Denmark.

*Air calorics* detects impairment of ipsilateral vestibular nerve fibers by slow thermal stimulation of primarily the lateral semicircular canal [7, 11]. We performed stimulation with sequential bithermal irrigation with air at 24 (cool) and 50 (warm) Celsius degrees. The Jongkees formula specified hypofunction; >25% unilateral weakness (UW) represented abnormal function [11–13]. In the study, the calorimetric response was recorded by the ICS Chartr 200 VNG/ENG by Otometrics, Natus, Taastrup, Denmark.

*Vestibular Evoked Myogenic Potentials* are short-latency, vestibular-dependent reflexes that are recorded from the sternocleidomastoid muscles in the anterior neck (cervical VEMPs – cVEMPs) and the inferior oblique extraocular muscles (ocular VEMPs – oVEMPs) [8]. For our study, we used the cVEMPs. The presence, the latency, and the amplitude of P1 and N1 waves were evaluated. If the waves were present bilaterally, the amplitude asymmetry ratio (Ars) was assessed. ARs > 0.35 (cVEMPs) were considered to indicate asymmetry between the two sides based on the normative values of our laboratory as well as data from the literature [8]. VEMPs Interacoustics A/S, Middelfart, Denmark did the recording.

*Video Head Impulse Test* is a video-oculography system that detects overt and covert saccades. It provides information about each semicircular canal function by quantifying the gain of the angular vestibulo-ocular reflex (aVOR) [7]. The normative range lies between 0.8 and 1.2 [14]. Our study uses the vHIT, EyeSeeCamvHIT, Interacoustics A/S, Middelfart, Denmark.

*Pure tone audiometry (PTA*) was used as a standardized evaluation of hearing function according to the Council on physical therapy - American medical association (CPT – AMA) guidelines [15], assessing hearing at four different frequencies (500 Hz/1 kHz/2 kHz/4 kHz). The average PTA hearing loss was then calculated for each patient.

### Questionnaires

*The Penn acoustic neuroma quality-of-life scale* is a valid disease-specific instrument for acoustic neuroma. It comprises 26 questions covering relevant domains such as hearing, balance, anxiety, energy, pain, and general quality of life. A higher score indicates a better quality of life [16].

*The Dizziness handicap inventory* was developed to evaluate the self-perceived handicapping effects of dizziness in everyday life imposed by vestibular system disease. It contains 25 items grouped into three content domains representing functional, emotional, and physical aspects of dizziness and unsteadiness. Possible scores range from 0 (no handicap) to 100 (severe handicap) [17].

*The Generalized Anxiety Disorder 7–Item Scale (GAD-7*) is a valid questionnaire for screening for generalized anxiety disorder (GAD) and assessing its severity. The seven items are scored from 0 to 3, so the GAD-7 scale score ranges from 0 to 21. Increasing scores on the scale are strongly associated with functional impairment [18].

Zung et al. [19] have developed a *self-rating depression scale (SDS)* to assess depression as a psychiatric disorder. The SDS consists of 20 items, each with a value of 1 to 4 depending on whether the item is worded positively or negatively, so the less depressed patient has the lower SDS score [19].

Neurotologists from the Department of Otorhinolaryngology and Head and Neck Surgery of the 1st Faculty of Medicine, Charles University in Prague, and Motol University Hospital developed the “*In-house questionnaire”*. It consists of nine questions about frequent complaints often seen in vestibular schwannoma patients (Table 1). Each has a score ranging from 1 to 4, with a higher score indicating a more severe handicap [20].

### Statistical analysis

As a drop-out is a naturally occurring phenomenon in longitudinal studies, we used multiple imputation procedures to replace missing values via the R-package Multiple Imputation by Chained Equations (MICE) [21]. We used predictive mean matching for continuous measures, multinomial logistic regression for categorical measures with more than two categories, and logistic regression for nominal measures. Given the relatively low level of missingness (11% of data were missing in total), we created a single imputed data set [22], which was used in subsequent analysis in SPSS version 25 (SPSS Inc., Chicago, IL, USA). Descriptive statistics of location and variability were then calculated for all variables in the study. The between-group differences (ITGG vs. VRG vs. CG) were examined using Kruskal-Wallis one-way analysis of variance (ANOVA), followed by post hoc comparison with the Mann-Whitney U test. The within-group differences of repeated measurements (e.g., time 1 vs. time 3) were analyzed by a Friedman test (an alternative to repeated-measures ANOVA), followed by a series of Wilcoxon signed-rank tests. Given the relatively small sample size for both between- and within-group comparisons, we used the Monte Carlo resampling procedure with *n* = 10.000 samples, which compensates for tied values and does not depend on asymptotic approximations for *p*-values [23]. Spearman correlation coefficient was used to examine bivariate associations between the variables. The level of statistical significance was set to α = 0.05.

## Results

### Demographics

The ITGG, VRG, and CG did not differ initially regarding demographic and clinical variables (age, gender, grade of tumor, canal paresis according to calorization, side of tumor) (all *p* > 0.05). All data are given in Online Resource 1.

### Prehabilitation

Intratympanic application of gentamicin led to a significantly larger aVOR gain reduction in all semicircular canals (all *p* < 0.05). Also, the UW in air calorics increased (*p* = 0.026) and the presence of cVEMPs decreased (*p* = 0.017) significantly due to chemical ablation. Before the gentamicin application, the average PTA was 64dB ± 20SD; after six to eight weeks, 4/7 patients suffered an ipsilesional increase in hearing impairment; the average PTA hearing loss was 18dB ± 5SD, 3/7 patient patients had PTA above 90 dB already at the baseline, and it did not change after the prehabilitation naturally.

### Questionnaires

#### DHI

**Fig. 2:**
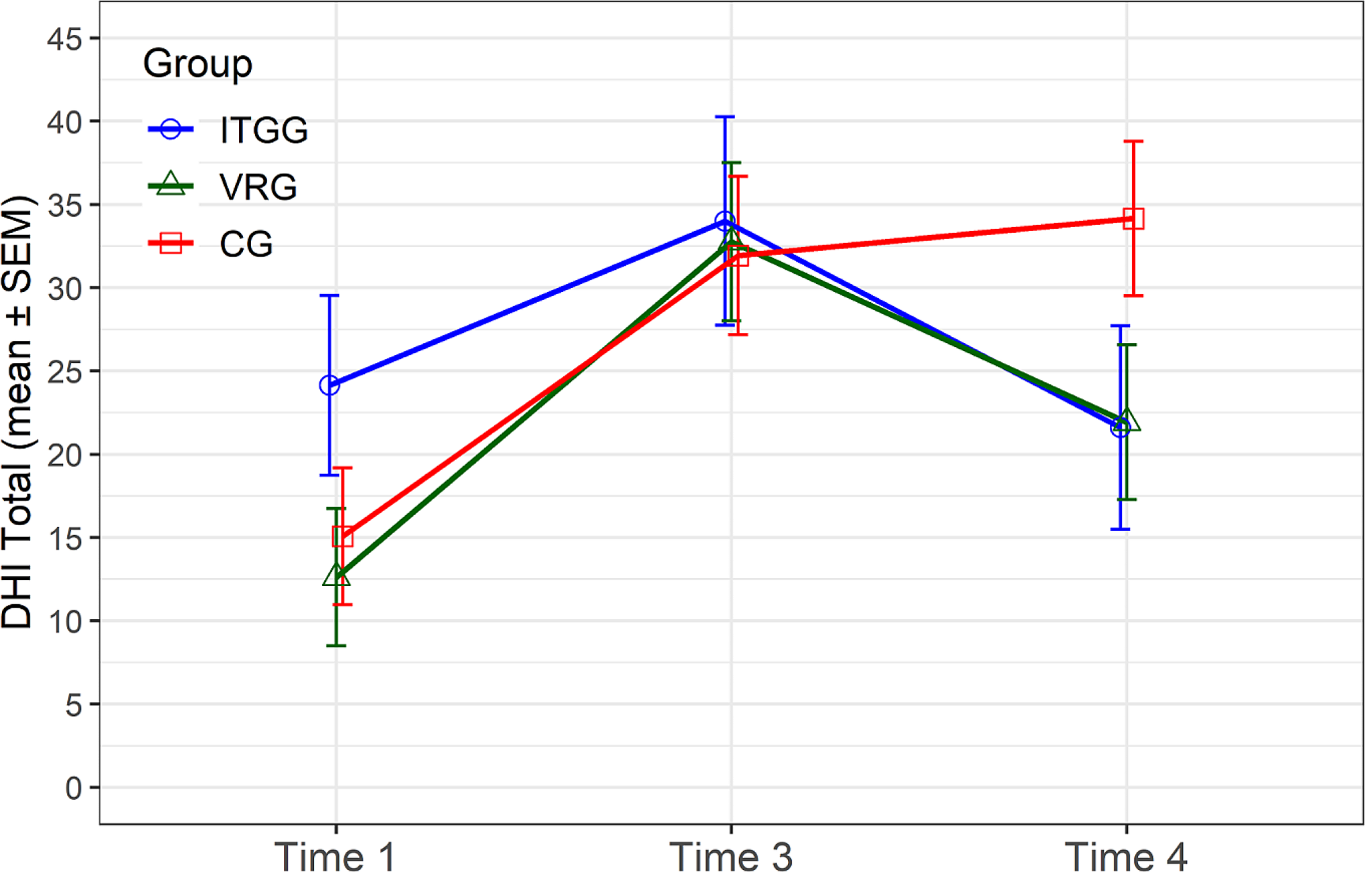
Dizziness handicap inventory

VRG showed significantly (*p* = 0.039) better outcomes in DHI than CG in a long-term follow-up (time 4) (Fig. 2). ITGG showed the same trends, too; however, *p*-values for this comparison were slightly above the selected significance level (*p* = 0.076). On the other hand, in the physical subscale of DHI, the results were significant for ITGG; it evinced significantly better scores than CG (*p* = 0.034). Also, VRG demonstrated a significantly better score than CG (*p* = 0.026) in the functional subscale of DHI.

#### In-house questionnaire

**Fig. 3:**
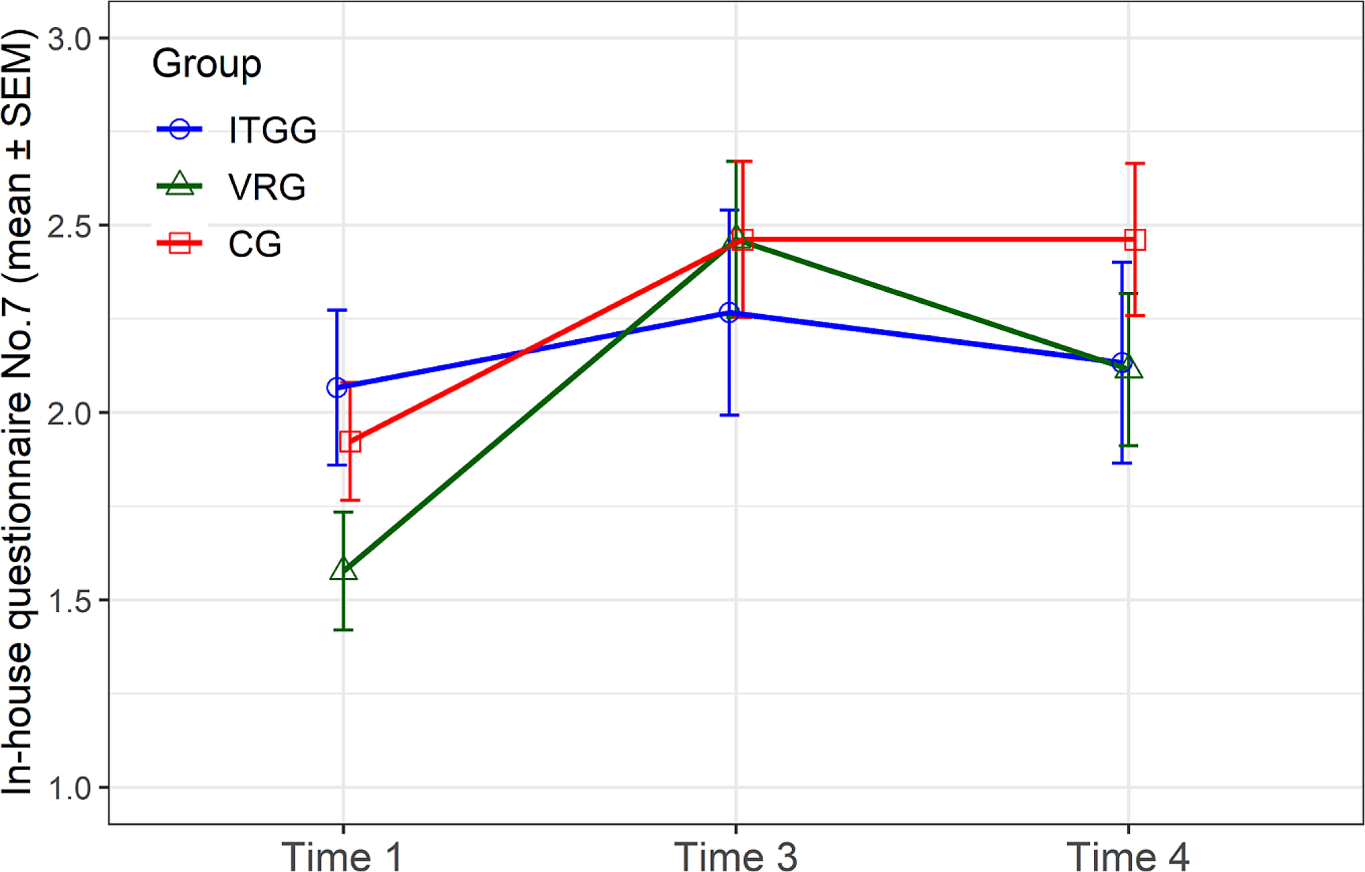
In-house questionnaire, question seven

Question seven in the In-house questionnaire considers patients’ sensitivity to complex visual and auditory stimuli. According to our results, exposure to virtual reality shortly after the surgery severely impacted a patient’s perception of complex stimuli (*p* = 0.002) (Fig. 3). However, in a long-term follow-up, the effect turned out to be positive, and the patients became more resilient to such an environment and felt more comfortable.

In GAD and PANQOL, our results showed no significant correlations or differences between the groups.

### Objective methods

#### vHIT

As expected, additional treatment did not affect aVOR gain on the operated or unoperated side. Based on these findings, we assume that vHIT is not a proper method for evaluating optokinetic stimulation in VRG or the effect of prehabilitation in ITGG in a long-term follow-up.

**Optokinetic testing**

**Fig. 4:**
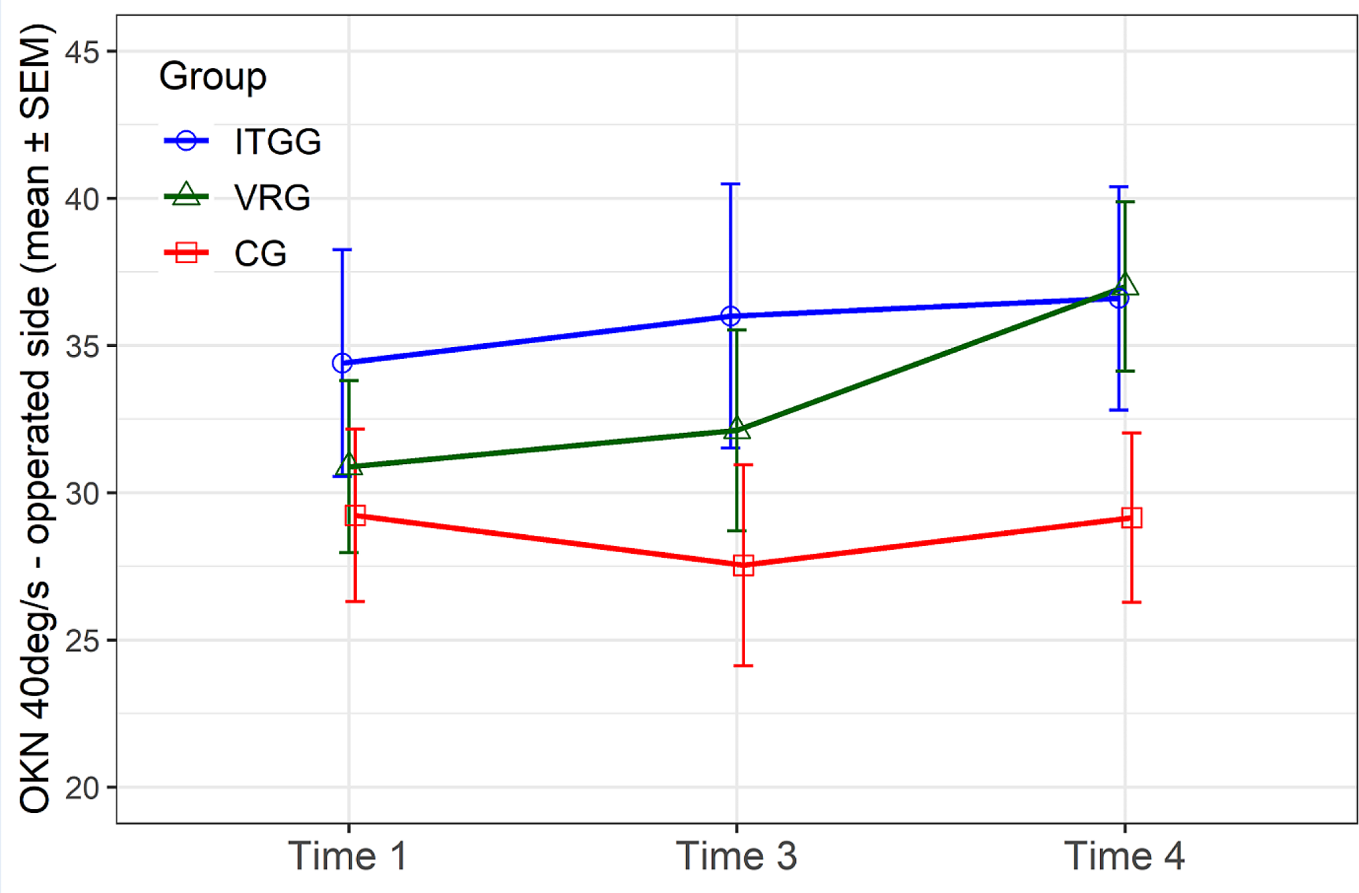
Optokinetic testing (velocity 40 deg/s, the operated side)

In optokinetic testing, for the speed of 40 deg/s and operated side, both targeted groups (ITGG and VRG) showed higher slow phase velocities (SPV) than the control group in a long-time follow-up (time 4) (Fig. 4); for ITGG, the mean value is 37 deg/s, median 38 deg/s ± 16SD, for VRG the mean value is 37 deg/s, median 36 deg/s ± 14 SD and for CG the mean value is 29 deg/s, median 29 deg/s ± 14SD.

Shortly after the surgery (time 3), in ITGG, there was a significant correlation between the vHIT lateral canal on the unoperated side and the Zung questionnaire (*r* = 0.67, *p* = 0.007) and In-house questionnaire No.6 and No.7 (*r* = 0,54, *p* = 0,039; *r* = 0,63, *p* = 0,011 respectively). There was also a correlation between vHIT of the lateral canal on the unoperated side in long-time follow-up (time 4) and DHI physical (*r* = 0,58, *p* = 0.025), In-house questionnaire No.3 (*r* = 0,57, *p* = 0.027), No.6 (*r* = 57, *p* = 0.028), No.7 (*r* = 71, *p* < 0.001).

Regarding VRG, in a long-time follow-up (time 4), the vHIT lateral canal on the unoperated side correlated with the DHI emotional (*r* = 0,48, *p* = 0.014) and the In-house questionnaire No.6 (*r* = 0,47, *p* = 0.022), No.7 (*r* = 0,43, *p* = 0.030), No.9 (*r* = 0,43, *p* = 0.029). We presume that the lesser decrease of vHIT gain on the unoperated side in ITGG and VRG may positively affect the patient’s perception of balance disorder and mood. No such correlation was observed in CG.

## Discussion

### Prehabilitation with gentamicin

Gentamicin is generally administered intravenously to treat various infections but is also used as an intratympanic injection for the suppression of the vestibular symptoms in Menier’s disease [24]. However, establishing the pharmacokinetics of gentamicin in the inner ear after local applications was challenging and this issue was addressed by several studies [25–27]. Gentamicin enters the perilymph of the inner ear independently through multiple pathways: (i) through the round window membrane (RWM) into the scala tympani (ST); (ii) through the oval window to the scala vestibuli (SV) and the vestibule. The studies verified the existence of substantial basal-apical gradients of gentamicin along the ST after the local application of the drug to the RWM and also demonstrated that the predominant vestibulotoxicity of locally applied gentamicin may arise from gentamicin’s distribution properties in the perilymph of the inner ear [25, 26]. On the other hand, the cochleotoxicity from intratympanic injections may result from the gentamicin level in SV rather than in ST, and the issue is far more complex and will need further study [27].

Importantly, vestibular prehabilitation with intratympanic gentamicin in patients with vestibular schwannoma could also be utilized for reducing the side effects of the iatrogenic lesions of the vestibular nerve. Its effectiveness in patients after schwannoma surgery is the subject of our current investigation. Due to the lack of data on patients with schwannoma, the 2017 Congress of Neurological Surgeons released a level-3 - recommendation (i.e., inconclusive evidence) on preoperative intratympanic gentamicin ablation to induce a controlled partial loss of semicircular canal function and to improve postoperative mobility [28]. According to *Tarnutzer et al.* [29, 30], the intratympanic application of gentamicin ends in relative sparing of the anterior semicircular canal, which is contrary to our study, where we managed to achieve high aVOR gain reduction even in the anterior semicircular canal. In 2023, reviews by *Potdar et al.* [31] and *Bassaletti et al.* [32] support this method but also raise some serious questions, namely the issue of long-term follow-up and a potential loss of hearing. Our study proved that the chemical ablation reduces aVOR gain in vHIT, increases the UW in air calorics, and decreases the presence of the cVEMP. Although the detailed pharmacokinetic properties of the locally applied gentamicin and its cochleotoxicity are incompletely resolved [25–27], it has been shown to be highly prevalent [33], which corresponds with our current observations. Therefore, the intratympanic gentamicin should be a contra-indication in cases of intended hearing preservation vestibular resection. These results substantiate our selection of the patients suitable for the prehabilitation. This finding is in agreement with the recent literature [34]. Regarding the long-term follow-up and subjective outcome, we provided evidence that the prehabilitated patients had significantly better results in the physical subgroup of DHI. The trend is similar but insignificant also in total DHI and optokinetic testing. In the case of PANQOL, which considers many perspectives of the quality of life in vestibular schwannoma patients, we believe that the results are affected by the postoperative functionality of the facial nerve to a large extent.

### Virtual reality

Customized vestibular rehabilitation, incorporating habituation, central sensory substitution, and tonic re-balancing at vestibular nuclei and other CNS levels, is currently considered a standard of care for patients with peripheral vestibular disorders regardless of age and symptom duration [34]. However, complete recovery is not usually achieved. According to *Abboud et al.* [35]., more than one out of four patients (28%) develop persistent symptoms of dizziness. One of the reasons underlying poor vestibular compensation appears to relate to the development of an over-reliance on visual input. It is known that all vestibular patients rely more on visual cues for postural stability [36, 37]. However, studies show that some patients are significantly more susceptible to visual motion than others [38, 39], which can lead to what is known as visual vertigo, visually induced dizziness, or, more recently, persistent postural perceptual dizziness (PPPD). These patients report a worsening or provocation of postural instability and vestibular symptoms in visually rich environments, such as crowds, moving traffic, escalators, or walking down supermarket aisles. For this reason, it is now postulated that in patients with visual vertigo symptoms, the treatment should include visual desensitization with repetitive visual-motion stimulation, e.g., exposure to optokinetic stimulation [36, 38–41. *Pavlou et al.* [40] 2012 further developed the idea of using dynamic virtual reality as an adjunct to a vestibular rehabilitation program for all patients with peripheral vestibular disorders. Recently, a smartphone-based gaming system for vestibular rehabilitation has been introduced [42]. It consists of two graded levels of difficulty that utilize optokinetic stimulation and discrete head movements in the pitch and yaw planes to achieve rehabilitation. This gaming system was a preliminary step in the development of an additional and more motivating treatment option for patients diagnosed with a peripheral vestibular disorder.

On the other hand, according to *Heffernan et al.* [43], there are already commercially available VR video games on the market that are congruent with standard vestibular rehabilitation. A relationship has been detected between the duration of the exposure to virtual reality environments and the magnitude of the therapeutic effects, suggesting that virtual reality should last at least 150 min of cumulated exposure to ensure positive outcomes [44]. Based on these findings, we hypothesized that in patients with acute unilateral peripheral loss (due to vestibular schwannoma resection), additional virtual reality-based 3D optokinetic stimulation in the early stage of central compensation might reduce the development of an over-reliance on visual cues.

Our data proved that VRG shows significantly better outcomes in DHI than CG in a long-term follow-up. The VRG also has better results in the in-house questionnaire, namely question No.7, considering the complex visual and auditory environments, and therefore, they are less prone to developing PPPD. Even though the results in optokinetic testing are insignificant, we presume that it could be associated with a better perception of visually complex surroundings as documented in the In-house questionnaire, question No.7. Another potential influencing factor that delays the entire recovery could be the level of physical activity. It is assumed that repetition of movement is needed to stimulate central vestibular compensation [45, 46]. Since the initial movements (and mainly the head movements) will provoke the vertigo symptoms due to the acute deafferentation [45] after the VS resection, the patients tend to alter the head movement strategy and lower variability in head movements during the gait tasks postoperatively [47, 48].

All the factors mentioned above can result in a vicious cycle of fear, avoidance of symptoms-provoking movements and situations, and increased handicap, retarding recovery even further [40, 49].

### Study limitations

Our main study limitation is the relatively small cohort size, which probably precluded us from reaching the statistical significance threshold in some endpoints.

However, given the incidence of vestibular schwannoma (1/100 000 person a year), the prospective cohort of 67 patients is considered a significant contribution to the literature.

We did not randomize all patients in our study cohort. Only patients with serviceable hearing were randomly divided into two study groups, as we describe in the methods section.

The long-term effect of virtual reality is unknown to date. In our study, we haven’t encountered any potential adverse effects while using virtual reality tools within a relatively short follow-up. As far as gentamicin prehabilitation is concerned, the main adverse effect is hearing loss, and we have not observed any other adverse effects in our study that could be related to gentamicin.

## Conclusions

Our study confirms that prehabilitation with gentamicin and postoperative virtual reality stimulation improves patients’ subjective perception of dizziness after vestibular schwannoma resection. Preoperative chemical ablation is a safe and effective procedure, but it is unsuitable in intended hearing preservation surgery cases. Virtual reality has no potential adverse effect. The challenge for further study is finding an objective test demonstrating virtual reality rehabilitation’s functional and complex impact on vestibular schwannoma patients.

## Electronic supplementary material

Below is the link to the electronic supplementary material.

Online Resource 1: Demographics (cVEMP – cervical vestibular myogenic-evoked potentials, vHIT – video head impulse test, Op – operated side, Unop – unoperated side, AAR – amplitude asymmetry ration, Op A – anterior semicircular canal of the operated side, Op L - lateral semicircular canal of the operated side, Op P - posterior semicircular canal of the operated side, Unop A - anterior semicircular canal of the unoperated side, Unop L - lateral semicircular canal of the unoperated side, Unop P - posterior semicircular canal of the unoperated side)
