# Supplementary figures and images for: The comparison between intratympanic gentamicin prehabilitation and postoperative virtual reality exposure to standard vestibular training in patients with vestibular schwannoma
Source: Eur Arch Otorhinolaryngol. 2024 Aug 10;282(1):79–89. doi: 10.1007/s00405-024-08891-8 (PMC11735475; doi:10.1007/s00405-024-08891-8)

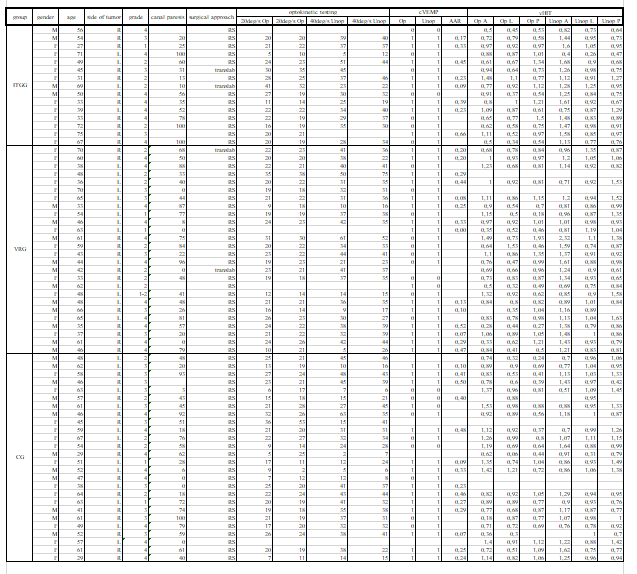

Supplement: Supplementary file 1 — Online Resource 1: Demographics (cVEMP – cervical vestibular myogenic-evoked potentials, vHIT – video head impulse test, Op – operated side, Unop – unoperated side, AAR – amplitude asymmetry ration, Op A – anterior semicircular canal of the operated side, Op L - lateral semicircular canal of the operated side, Op P - posterior semicircular canal of the operated side, Unop A - anterior semicircular canal of the unoperated side, Unop L - lateral semicircular canal of the unoperated side, Unop P - posterior semicircular canal of the unoperated side) [file 405_2024_8891_MOESM1_ESM.png]
